# Do Work Characteristics Predict Health Deterioration Among Employees with Chronic Diseases?

**DOI:** 10.1007/s10926-017-9716-z

**Published:** 2017-06-29

**Authors:** Astrid de Wind, Cécile R. L. Boot, Ranu Sewdas, Micky Scharn, Swenne G. van den Heuvel, Allard J. van der Beek

**Affiliations:** 1 Department of Public and Occupational Health, Amsterdam Public Health research institute, VU University Medical Center, Van der Boechorststraat 7, 1081 BT Amsterdam, The Netherlands; 2 Body@Work, Research Center on Physical Activity, Work and Health, TNO-VU/VUmc, Amsterdam, The Netherlands; 3 Work Health Technology, Netherlands Organisation for Applied Scientific Research TNO, Schipholweg 77-89, 2316 ZL Leiden, The Netherlands

**Keywords:** Chronic disease, Work characteristics, Health deterioration, Employees, Longitudinal study

## Abstract

*Purpose* In our ageing workforce, the increasing numbers of employees with chronic diseases are encouraged to prolong their working lives. It is important to prevent health deterioration in this vulnerable group. This study aims to investigate whether work characteristics predict health deterioration over a 3-year period among employees with (1) chronic diseases, and, more specifically, (2) musculoskeletal and psychological disorders. *Methods* The study population consisted of 5600 employees aged 45–64 years with a chronic disease, who participated in the Dutch Study on Transitions in Employment, Ability and Motivation (STREAM). Information on work characteristics was derived from the baseline questionnaire. Health deterioration was defined as a decrease in general health (SF-12) between baseline and follow-up (1–3 years). Crude and adjusted logistic regression analyses were performed to investigate prediction of health deterioration by work characteristics. Subgroup analyses were performed for employees with musculoskeletal and psychological disorders. *Results* At follow-up, 19.2% of the employees reported health deterioration (N = 1075). Higher social support of colleagues or supervisor predicted health deterioration in the crude analyses in the total group, and the groups with either musculoskeletal or psychological disorders (ORs 1.11–1.42). This effect was not found anymore in the adjusted analyses. The other work characteristics did not predict health deterioration in any group. *Conclusions* This study did not support our hypothesis that work characteristics predict health deterioration among employees with chronic diseases. As our study population succeeded continuing employment to 45 years and beyond, it was probably a relatively healthy selection of employees.

## Introduction

Many developed countries are confronted with ageing populations, which puts pressure on social security systems [1]. Several governments responded to the changing composition of the population by reforms to reduce early exit from the workforce and stimulate prolonged working [1]. These measures are aimed at encouraging older workers to prolong their working lives. The increase of older workers in the workforce is likely to have consequences for the health composition of the workforce as well; as the prevalence of chronic diseases increases with age, it is likely that the workforce will consist of a larger proportion of persons suffering from chronic diseases. In fact, in 2013, 58% of the Dutch population aged 50–55 years and 66.2% of the population aged 55–65 years reports having at least one chronic disease [2]. At the same time, accessibility to disability pension has been decreased in the Netherlands. These developments in social security may lead to increasing numbers of employees with chronic diseases in the workforce in the near future.

Employees with chronic diseases who previously may have left the workforce by becoming work disabled, unemployed or retiring early, may prolong their working lives, because of reduced possibilities to leave work. In general, work is good for health. A recent review showed that employment reduces the risk of depression and improves general mental health [3]. However, this does not necessarily mean that working has beneficial health effects for everyone in every situation. In fact, a previous study provided indications that certain working conditions may worsen health in employees with chronic diseases, since it has been found that the association between health and sickness absence is partially explained by work characteristics [4]. Employees with chronic diseases may, in general, be more vulnerable for the negative effects of adverse work characteristics compared to the healthy part of the working population. As working with a chronic disease will become more common, especially among older workers, it is important to gain insight into the course of health while working with a chronic disease until higher age. Even though chronic diseases are difficult to eliminate, the work environment may contribute to prevention of health deterioration.

Although previous studies have provided insight into the implications of having a chronic disease on sickness absence [5], work ability and productivity at work [6], and early exit from work [7, 8], it is unclear how perceived health of employees with chronic diseases may develop and which work characteristics may negatively impact the health course of employees with chronic diseases. Gaining more insight into this may improve our knowledge on which work-related interventions have the potential to optimally support employees with chronic diseases to maintain their health. Certain chronic diseases, such as musculoskeletal or psychological disorders, are more strongly related to work than other chronic diseases, such as diabetes mellitus or circulatory disorders. To illustrate, previous reviews showed that physical work demands, i.e. lifting heavy loads, excessive repetitive movements and awkward postures, predict musculoskeletal disorders [9, 10]. In addition, adverse psychosocial work characteristics, i.e. higher emotional demands and low support at work, have been found to predict poor mental health [11–13]. Musculoskeletal and psychological disorders also predicted early retirement in previous research, whereas other chronic diseases did not [8]. To the knowledge of the authors, the influence of work characteristics on the health course of employees with chronic diseases, and more specific with musculoskeletal and psychological disorders has not been studied before. To contribute to these gaps in the literature, this study aims to investigate whether work characteristics predict health deterioration over a 3-year period among employees with chronic diseases. The second aim is to investigate whether work characteristics predict health deterioration among employees with musculoskeletal and psychological disorders.

## Methods

### Data and Study Population

Data of the Study on Transitions in Employment, Ability and Motivation (STREAM) were used. STREAM is a Dutch longitudinal study among 15,118 persons, including 12,055 employees, 1029 self-employed persons, and 2034 persons without paid employment aged 45–64 years. These persons filled out a yearly online questionnaire from 2010 to 2013 and since 2015. Questionnaires consisted of questions about, among others, work, health and employment status. The study population and measurements of STREAM have previously been described elsewhere [14]. In the present study, we used data of the first four waves of STREAM.

Inclusion criteria for the present study were being an employee during at least two measurements (baseline and one of the follow-up measurements in 2011, 2012 or 2013), having at least one chronic disease at baseline and complete information on general health (i.e. in the baseline and follow-up questionnaire). Information on being an employee was derived from one question asking persons to indicate their employment status, with, among others, the answering options ‘a paid job’ and ‘multiple paid jobs’. Those who indicated that they had one or more paid jobs were considered being an employee and thus included in the study population. Presence of a chronic disease was assessed using the following question: ‘Do you (currently) have one or more of the following chronic diseases, disorders or handicaps?’. This question had the following answering options: problems with hand and arms, problems with legs and feet, problems with back and neck, severe headache or migraines, circulatory disorders, respiratory disorders, digestive disorders, diabetes mellitus, problems with skin, psychological disorders, problems with hearing, epilepsy, life threatening illnesses, problems with vision, and other chronic diseases, disorders or handicaps. Those who indicated to have at least one of these chronic diseases were included in the study population. Persons reporting the score ‘poor’ on the general health question of the SF-12 [15] at baseline were excluded, as we could not measure deterioration in this group due to floor effects. These in- and exclusion criteria resulted in a study population of 5600 persons.

With regard to the second research aim only those employees who had musculoskeletal or psychological disorders were included in the analyses. Problems with hands and arms, problems with legs and feet and problems with back and neck were merged into one group with musculoskeletal disorders.

### Measurements

Information on work characteristics and potential confounders was derived from the baseline questionnaire. Information on general health was derived from the questionnaires at baseline and the follow-up measurements.

#### Outcome: Health Deterioration

General health was assessed using the following question from the SF-12 [15]: ‘How is your health in general?’ This question could be answered according to the following answering options: ‘excellent’, ‘very good’, ‘good’, ‘moderate’, and ‘poor’. Health deterioration was defined as having a lower score on this question during follow-up than at baseline. Which follow-up measurement was used to assess health deterioration, was based on the last measurement in which someone was an employee. This may be the last year of follow-up, the year before someone exited from the workforce, or the year before someone did not participate in the study.

#### Predictors: Work Characteristics

With regard to work characteristics, four types of demands (physical, mental, emotional and psychological work demands) and two types of resources (social support of colleagues and supervisor and autonomy) were included. The items on the six work characteristics all had a five-point answering scale ranging from ‘(almost) never’ to ‘always’. Physical demands at work were measured using five items on force exertion, static load and vibrations (Cronbach’s alpha 0.86) based on the Netherlands Working Conditions Survey [16] and the Dutch Musculoskeletal Questionnaire [17]. An example item is ‘Do you work in awkward postures?’. Mental demands were measured using slightly adjusted questions derived from NOVA-WEBA (Cronbach’s alpha 0.78) [18]. An example item of mental demands is ‘Does your work require intensive thinking?’. Emotional demands were measured using three questions derived from the Copenhagen Psychosocial Questionnaire (Cronbach’s alpha 0.86) [19]. An example item of emotional demands is ‘Do you become emotionally involved in your work?’. Psychological job demands were measured using four items derived from the Job Content Questionnaire (Cronbach’s alpha 0.87) [20]. An example item of psychological job demands is ‘Do you have to work very fast?’. Autonomy was measured using five items, also derived from the Job Content Questionnaire (Cronbach’s alpha 0.79). An example item is ‘Are you able to decide for yourself how to do your work?’. Social support of colleagues and supervisor was measured using four items derived from the Copenhagen Psychosocial Questionnaire (Cronbach’s alpha 0.82) [19]. An example item is ‘How often do you get help and support from your immediate supervisor?’. All predictors were inspected on their distributions. If distributions were skewed, they were dichotomized distinguishing between the most adverse quartile versus the other quartiles combined (reference group). If predictors were normally distributed they were kept as continuous variables in the analyses.

#### Confounders

Age, gender, educational level, work adjustments, baseline general health and comorbidity were included as confounders in the analyses. Educational level was measured using a question on the highest level of education completed with a diploma, and categorized into low (primary school, lower and intermediate secondary education, or lower vocational training), intermediate (higher secondary education, or intermediate vocational training) or high (higher vocational education or university). With regard to work adjustments, employees indicated whether different facilities for older workers, including the possibility for adjustments in the workplace due to health problems, and adjustments in work tasks due to health problems were available and whether they used them. The two questions were combined and dichotomized into ‘did not use work adjustments’ and ‘used work adjustments’. Baseline general health was based on the previously mentioned question regarding general health. Answers were dichotomized, distinguishing between good (‘excellent’, ‘very good’, or ‘good’) and moderate health (‘moderate’). Comorbidity was assessed based on the previously mentioned question on chronic diseases, disorders or handicaps, i.e. those who indicated to have more than one of the options were considered having comorbidity. Note that problems with hands and arms, problems with legs and feet and problems with back and neck were considered to be one chronic disease.

### Analyses

Descriptive statistics, i.e. means, standard deviations, frequencies, and percentages, were used to report on baseline characteristics, i.e. work characteristics, health and potential confounders. To check whether lost to follow-up was selective, we compared baseline characteristics of those who were lost to follow-up to those of the participants by means of independent T-tests.

Predictors of health deterioration were studied by logistic regression analyses. Odds ratios (OR) and 95% confidence intervals (95% CI) were calculated. Analyses with regard to the first research aim, i.e. investigating prediction of health deterioration by work characteristics among employees with chronic diseases, took place in three steps. In the first step of the analyses, crude logistic regression analyses were established for all work characteristics separately. Second, adjusted logistic regression analyses including confounders were performed for all work characteristics separately. In the third step interaction terms between general health (dichotomous) and work characteristics were included to the multivariate adjusted models of the second step. If the interaction terms revealed to be significant (p < 0.05), subgroup analyses were planned for a group of persons reporting moderate health and a group of persons reporting good health. Distinction in these two groups was based on the general health score at baseline; those indicating that their health was ‘excellent’, ‘very good’ or ‘good’ were considered as having good health and the group moderate health consisted of persons indicating their health as ‘moderate’. As previously described, persons indicating that their health was ‘poor’ were excluded, as we could not measure a health deterioration in this group due to floor effects. For the second research aim focusing on employees with musculoskeletal and psychological disorders, steps 1 and 2 were repeated for these subgroups. Analyses were performed using SPSS Statistics version 22 (SPSS Institute, Cary NC, USA).

#### Sensitivity Analyses

An alternative way of defining health deterioration would have been a deterioration to poor health instead of any health deterioration. This alternative may provide insight into a more clinically relevant health deterioration. To test whether this alternative also yields other results with regard to predictors, we conducted sensitivity analyses defining health deterioration as a deterioration in general health from either ‘excellent’, ‘very good’, ‘good’ or ‘moderate’, to ‘poor’.

### Ethical Issues

The Medical Ethical Committee of the VU University Medical Center Amsterdam, the Netherlands, declared that the Dutch Medical Research Involving Human Subjects Act does not apply to STREAM. The Medical Ethical Committee had no objection to the execution of this study. In the information for participants that accompanied the online questionnaires, it was emphasized that the privacy of participants was guaranteed, all answers to the questions were treated confidentially, and all data were stored in secured computer systems. By filling in the questionnaire, participants implicitly gave permission for the use of the data.

## Results

Table 1 shows baseline characteristics of the total study population. Of this group, 3093 persons reported musculoskeletal disorders and 345 persons reported psychological disorders. In total, 1075 persons (19.2%) reported a deterioration of health between the baseline and follow-up measurement. At baseline, persons lost to follow-up reported somewhat higher psychological job demands than participants included in our study (3.2 vs. 3.1) (data not shown). There were no other significant differences between participants and persons lost to follow-up.

**Table 1:** Baseline characteristics of the total study population (N = 5600)

Characteristic	Unit, categories, range	Mean (N)	SD (%)
Age	Years	54.2	5.2
Gender	Female	2636	47%
Educational level	Low	1550	28%
	Intermediate	2243	40%
	High	1807	32%
Work adjustments	Yes	455	8%
Health			
Poor general health (d)	Yes	1160	21%
Musculoskeletal disorders	Yes	3093	55%
Psychological disorders	Yes	345	6%
Comorbidity	Yes	2576	46%
Predictors			
High physical demands at work (d)	Yes	1435	26%
High mental demands at work (d)	Yes	1194	21%
High emotional demands at work (d)	Yes	2252	40%
Psychological job demands (c)	1–5	3.2	0.8
Social support colleagues/supervisor (c)	1–5	3.6	0.8
Autonomy (c)	1–5	3.8	0.7

*d* dichotomous, *c* continuous

### Prediction of Health Deterioration by Work Characteristics

The crude analyses revealed that employees reporting more social support of colleagues or supervisor were more likely to report health deterioration at follow-up (OR 1.11, 95% CI 1.01–1.21) (Table 2). However, this effect was attenuated in the adjusted analyses. The other work characteristics did not predict health deterioration, neither in the crude nor in the adjusted analyses. Psychological job demands showed a tendency towards significance (p < 0.10), suggesting that employees reporting higher psychological job demands were more likely to report health deterioration during follow-up (OR 1.09, 95% CI 0.99–1.19). None of the interaction terms, i.e. between the separate work characteristics and baseline health, were statistically significant.

**Table 2:** Results of logistic regression analyses: prediction of health deterioration by work characteristics among employees with chronic diseases (N = 5600), musculoskeletal disorders (N = 3093) and psychological disorders (N = 345)

Characteristic	Categories, range	Crude models						Adjusted models^a^					
		Total group		Musculoskeletal disorders		Psychological disorders		Total group		Musculoskeletal disorders		Psychological disorders	
		OR	95% CI	OR	95% CI	OR	95% CI	OR	95% CI	OR	95% CI	OR	95% CI
High physical demands at work (d)	Yes	0.99	0.85–1.15	0.85	0.69–1.05	1.35	0.75–2.42	1.09	0.92–1.29	0.96	0.76–1.20	1.21	0.62–2.37
High mental demands at work (d)	Yes	1.09	0.93–1.28	1.20	0.97–1.48	1.22	0.67–2.25	1.10	0.92–1.32	1.18	0.93–1.49	1.84**	0.92–3.69
High emotional demands at work (d)	Yes	0.95	0.83–1.09	0.88	0.73–1.05	0.90	0.53–1.53	1.07	0.92–1.25	0.95	0.77–1.16	1.04	0.58–1.87
Psychological job demands (c)	1–5	1.05	0.96–1.14	1.02	0.91–1.15	1.02	0.73–1.41	1.09**	0.99–1.19	1.08	0.95–1.23	1.23	0.86–1.77
Social support colleagues/supervisor (c)	1–5	1.11*	1.01–1.21	1.15*	1.02–1.29	1.42*	1.01–1.99	0.99	0.90–1.09	1.10	0.97–1.25	1.13	0.78–1.63
Autonomy (c)	1–5	1.07	0.97–1.17	1.06	0.93–1.20	1.11	0.76–1.62	0.96	0.86–1.06	0.93	0.81–1.07	1.13	0.75–1.71

*d* dichotomous, *c* continuous

*Significant at p < 0.05

**Significant at p < 0.10

^a^Adjusted for age, gender, educational level, previous work adjustments, baseline health and comorbidity

### Prediction of Health Deterioration by Work Characteristics Among Employees with Musculoskeletal and Psychological Disorders

The crude analyses in the group with musculoskeletal disorders and the group with psychological disorders revealed that social support of colleagues or supervisor was the only work characteristic that predicted health deterioration during follow-up (Table 2). Employees who reported more social support were more likely to report health deterioration, with an OR of 1.15 (95% CI 1.02–1.29) for employees with musculoskeletal disorders and an OR of 1.42 (95% CI 1.01–1.99) for employees with psychological disorders. These effects were attenuated after adjustment for confounders. The adjusted analyses among employees with psychological disorders revealed that mental demands showed a tendency towards significance (p < 0.10) indicating that high mental demands predicted health deterioration (OR 1.84 95% CI 0.92–3.69). The other work characteristics did not predict health deterioration in any of these two groups.

### Sensitivity Analyses

Using the alternative definition of health deterioration, i.e. deterioration to poor health did not lead to major differences compared to the main analyses. In contrary to the main analyses, the sensitivity analyses showed that high mental demands at work predicted health deterioration to poor health, both in the crude and adjusted analyses, in the total group, the group with musculoskeletal health problems and the group with psychological disorders (Appendix).

## Discussion

This study did not support our hypothesis that work characteristics predict health deterioration among employees with chronic diseases over a 3-year period. However, the results suggested that high mental demands predicted health deterioration among employees with psychological disorders.

We found no indications that work characteristics predict health deterioration among employees with chronic diseases. This seems not in line with research using the same cohort data, that showed that several adverse work characteristics were associated with poorer mental and physical health among employees with and without chronic diseases [21]. However, mixed findings might be explained by differences in the outcome measure, i.e. general health versus mental and physical health, and the study population, i.e. employees with a chronic disease as opposed to employees with or without a chronic disease. Finally, effect sizes of this previous study were small. Moreover, our health deterioration measure may be more conservative than the previously used mental and physical health measures, as it is more susceptible for floor (and ceiling) effects and allows for less variation.

With regard to musculoskeletal disorders, we expected especially physical work demands to be an important predictor of health deterioration. This seems to be a logical consequence of findings from previous research that showed that physical work demands contribute to musculoskeletal disorders [9, 10]. However, we did not find indications that physical work demands contribute to health deterioration during follow-up. This challenges the existing belief that certain working conditions may worsen health in employees with chronic diseases. However, it should be acknowledged that our study population of workers with chronic diseases was a healthy selection of the total population with chronic diseases, as they had managed to continue participation in paid work until older age, i.e. a healthy worker effect. This group might have settled with their health problems, and adjusted their work environment to match their abilities and limitations resulting from their disease. For example, employees with musculoskeletal disorders might have left jobs with high exposure to physical demands, and eventually exchanged these jobs for more mentally demanding jobs that better matched their physical abilities. Among employees with psychological disorders those reporting high mental demands seemed to experience health deterioration more often. This is in line with our expectation and matches previous research showing that adverse psychosocial work characteristics predict poor mental health [11–13]. We did not find indications for prediction of health deterioration by the other work characteristics, which may be explained by the healthy worker effect as described previously.

Surprisingly, the crude findings with regard to support of colleagues or supervisor among all groups are opposite to what we would have expected, with the strongest counter-intuitive finding among employees with psychological disorders. In line with this finding, a previous study showed that perceiving more support from the employer predicted sickness absence after 1 year [22]. It might be reasoned that employees start to know how supportive their working environment is from the moment they need it. Our measure of social support may reflect support to persons experiencing chronic diseases that may result in the health deterioration itself.

### Methodological Considerations

To the best of our knowledge this is the first study to investigate the influence of work characteristics on health deterioration among older employees with chronic diseases over a 3-year follow-up period. As people, also those with a chronic disease, are largely encouraged to prolong their working lives it is of interest to focus on the potential of the work environment to prevent health deterioration among this group of workers. The analyses were conducted using a large dataset, which allowed us to investigate a variety of work characteristics, including both job demands and job resources. In addition, we performed longitudinal analyses by investigating health deterioration over a 3-year period. Furthermore, it allowed us to adjust for several confounders and to perform subgroup analyses among employees with musculoskeletal or psychological disorders. Finally, the analyses showed that operationalization of health deterioration matters. We defined health deterioration as any deterioration in general health between baseline and follow-up on a 5-point scale. The analyses using this definition did not support our hypothesis that work characteristics predict (any) health deterioration among employees with chronic diseases (except for a tendency towards significance among employees with psychological health problems). However, sensitivity analyses with a more strict definition of health deterioration, indicated that high mental demands at work predicted deterioration to poor health among all groups. In future research it is thus important to carefully consider what kind of outcome one is interested, as different definitions may change contrasts resulting in different findings.

A limitation is that work characteristics may have already been adjusted at the workplace according to one’s specific health situation before the baseline measurement. As speculated above, this may have resulted in a biased study population containing of relatively healthy employees, albeit suffering from chronic disease. As work limitations resulting from chronic diseases may have resulted in adjustments in the work environment, this complicates disentangling the influence of work characteristics on general health. However, in the analyses we controlled for work adjustments. Moreover, previous research showed that work adjustments are often implemented after longer periods of sick leave, which indicates that work adjustments are implemented mainly following serious productivity losses [23]. Even though we controlled for work adjustments, we cannot rule out that our associations are an underestimation of the true effects in a population with a less optimal work environment. A second limitation is that all data relied on self-reports. With regard to chronic diseases, it is not sure whether employees indeed were diagnosed with these diseases and whether all diagnosed diseases were reported. A disadvantage of self-reported diagnosis is that both under and over reporting may take place. However, previous studies have shown that the reliability of self-reports is acceptable. In addition, as we were interested in relations between work characteristics and health deterioration within groups of workers with chronic disease, we do not expect, a major bias because of this.

### Future Research

As previously said, we expect that our study population was a relatively healthy selection of employees with a chronic disease aged 45 years and up. They succeeded in continuing their working careers to 45 years and beyond, whereas the more vulnerable employees with a chronic disease may already have left paid employment before reaching this age. In this population of older workers we found no indications that work characteristics predict health deterioration. It is of interest, however, whether the work environment may contribute to prevention of health deterioration among younger employees with a chronic disease, and by doing so, lengthen the time they are active in paid employment. Therefore, future research is recommended to investigate the influence of work characteristics on health deterioration among younger employees (<45 years). Furthermore, it is of interest to pay attention to health improvement in addition to health deterioration. This may give insight into favorable working conditions that may improve health. As employees without chronic disease may not feel healthy, comparing health trajectories of employees with chronic diseases and employees without chronic diseases may provide additional insights here.

Furthermore, it should be noted that at the time of data collection possibilities to leave the workforce early were still widely accessible. Declining accessibility to disability pension as well as to favorable early retirement schemes, may probably increase the proportion of people with chronic diseases remaining active in the workforce. This, in turn, may increase the importance of a favorable work environment. Future research among older employees with chronic diseases is needed to explore the potential of the work environment in the prevention of health deterioration in future generations.

## Conclusion

Our study did not support our hypothesis that work characteristics predict health deterioration among employees with chronic diseases. However, the results suggest that among employees with psychological disorders high mental demands at work predict health deterioration. Thus, in the development of policy measures aiming to prevent health deterioration and to enhance a sustainable working life among employees with chronic diseases, diversity in this group should be taken into account.

## Appendix

See Table 3.

**Table 3:** Results of logistic regression analyses (sensitivity analyses): prediction of health deterioration by work characteristics among employees with chronic diseases (N = 5600), musculoskeletal disorders (N = 3093) and psychological disorders (N = 345)

Characteristic	Categories, range	Crude models						Adjusted models^a^					
		Total group		Musculoskeletal disorders		Psychological disorders		Total group		Musculoskeletal disorders		Psychological disorders	
		OR	95% CI	OR	95% CI	OR	95% CI	OR	95% CI	OR	95% CI	OR	95% CI
High physical demands at work (d)	Yes	1.25	0.82–1.91	1.28	0.78–2.11	1.21	0.45–3.24	1.17	0.75–1.84	1.22	0.72–2.07	1.29	0.43–3.88
High mental demands at work (d)	Yes	1.66*	1.09–2.52	1.61**	0.97–2.67	3.50*	1.40–8.74	1.71*	1.10–2.65	1.56	0.92–2.65	5.76*	1.98–16.74
High emotional demands at work (d)	Yes	0.98	0.66–1.46	1.00	0.63–1.61	1.16	0.47–2.88	0.92	0.66–1.40	0.95	0.58–1.57	1.31	0.49–3.49
Psychological job demands (c)	1–5	1.27**	0.98–1.64	1.30**	0.96–1.76	1.54	0.87–2.73	1.25**	0.97–1.61	1.28	0.95–1.74	1.30	0.70–2.43
Social support colleagues/supervisor (c)	1–5	1.07	0.83–1.37	0.99	0.73–1.34	0.99	0.58–1.69	1.25**	0.96–1.63	1.12	0.82–1.53	1.03	0.56–1.87
Autonomy (c)	1–5	0.87	0.67–1.14	0.91	0.66–1.25	1.00	0.53–1.88	1.03	0.78–1.35	1.05	0.75–1.48	1.38	0.72–2.67

*d* dichotomous, *c* continuous

*Significant at p < 0.05

**Significant at p < 0.10

^a^Adjusted for age, gender, educational level, previous work adjustments, baseline health and comorbidity
